# Ground User Clustering for Adaptive Multibeam GEO Satellite Networks

**DOI:** 10.3390/s26082384

**Published:** 2026-04-13

**Authors:** Heba Shehata, Hazer Inaltekin, Iain B. Collings

**Affiliations:** Faculty of Science and Engineering, Macquarie University, Sydney, NSW 2109, Australia; heba.shehata@mq.edu.au (H.S.); iain.collings@mq.edu.au (I.B.C.)

**Keywords:** adaptive multibeam satellite systems, GEO satellite communications, user clustering, beam placement optimization, beam hopping, geometric set cover, phased-array antennas

## Abstract

This paper considers geometry-aware ground user clustering and Cluster Center Optimization for beam pointing and scheduling in adaptive multibeam Geostationary Earth Orbit (GEO) satellite networks. By grouping ground users, beams can be directed toward user clusters to maximize satellite throughput. We propose GeoClust, a polynomial-time geometric user clustering algorithm for adaptive multibeam GEO satellite networks, using a geometric set-cover approach that explicitly balances link signal-to-interference-plus-noise ratio (SINR) and hopping overhead. The algorithm employs a Boyle–Dykstra projection-based cluster center update within an alternating optimization framework, combined with nearest-center membership updates, to enforce the cluster-radius constraint while ensuring feasibility and provable convergence. It also achieves near-linear throughput scaling with increasing number of RF chains. Numerical evaluations on real-world population data show that, under heavy traffic conditions, our approach more than doubles the zero outage and median user rates compared to benchmark schemes.

## 1. Introduction

Recent progress in multibeam Geostationary Earth Orbit (GEO) satellites equipped with phased-array antennas is accelerating broadband delivery to remote areas [1,2]. The fixed geometry of GEO systems supports persistent regional coverage and effective mobility management (relative to non-geostationary constellations), and the adaptive antennas enable on-demand formation of many narrow spot beams over wide service areas [3]. System performance is fundamentally governed by the distribution of ground users, and the process by which they are clustered/grouped and spatially multiplexed, to access limited radio resources [4,5]. Phased-array antennas enable the dynamic generation and steering of multiple narrow spot beams [6,7] and can support multi-gigabit aggregate throughputs [8,9,10].

In practice, spatial multiplexing is realized through beam hopping in the temporal domain and frequency reuse in the spectral domain. Both mechanisms require careful control of the spatial overlap and separation of simultaneously illuminated regions to manage inter-beam interference (IBI) [11,12,13]. This makes user clustering and beam footprint placement tightly coupled design problems.

This paper develops a low-complexity clustering algorithm that explicitly balances cluster compactness against the number of clusters, thereby accounting for both IBI-limited spatial reuse and beam-hopping overhead. A key methodological contribution is a projection-based alternating optimization framework in which Boyle–Dykstra updates enforce radius feasibility during cluster center refinement. The proposed method admits provable convergence guarantees and achieves strong empirical performance on realistic deployment scenarios.

Existing approaches include fixed beam placement techniques, which tessellate the service area with pre-planned cells and, by construction, avoid explicit user clustering [14,15,16,17,18,19]. However, fixed layouts cannot adapt to traffic non-uniformity, leading to persistent resource misallocation where some beams are underutilized while others are demand-starved.

The beam placement approaches in [20,21] attempt to accommodate traffic non-uniformity through beam partitioning and densification techniques. Those methods extend fixed beam architectures by incorporating cell-splitting and frequency-slicing to better match user demand variations. However, they still operate within a fixed beam layout and do not consider the core problem of user clustering to dynamically group users into a small number of geographically compact clusters for beam allocation. As such, their adaptability remains limited, falling short of fully leveraging the dynamic beamforming capability of phased-array antennas.

Cluster-based beam placement optimization is an approach where ground users are first clustered, and spot beams are subsequently directed toward the clusters to maximize the satellite throughput. The user clustering step can be posed as a geometric set cover problem, which is NP-hard [22,23,24]. Consequently, a range of approximate and heuristic methods have been investigated in multibeam satellite communications [25,26,27,28,29,30,31].

A multicast grouping scheme was presented in [25], which aimed to maximize average data rates for a given number of beams. In [26,27,28], graph-theoretic approximations, such as edge-clique cover and *p*-center formulations have been proposed to reduce the problem to more tractable combinatorial structures. Meta-heuristics presented in [29,30], including genetic algorithms and self-organizing maps combined with *K*-means clustering, search the solution space stochastically. The work in [31] presents deterministic geometric heuristics, such as density-based methods, forming user clusters sequentially, starting from the densest regions. Related throughput optimization work also considers joint beamforming and scheduling decisions, often in LEO networks, via optimization and learning-based formulations [32,33].

Maximizing satellite throughput requires jointly considering beam placement and beam scheduling. From the clustering perspective, throughput optimization depends on a fundamental trade-off between the size and number of clusters. Forming too few clusters leads to geographically dispersed user groups, resulting in reduced user signal-to-interference-plus-noise ratios (SINRs) and inefficient RF chain utilization under beam-hopping schedules [34].

Conversely, forming too many small clusters increases the number of hops required to serve all users within a beam-hopping window [1,34,35]. Each hop incurs system-level overhead, including beam switching time (for updating beam parameters), guard time (for synchronization), and processing time (for link adaptation and resource scheduling), which typically ranges from tens to several hundred microseconds [36,37] (We model these non-data overhead components by an effective per-hop overhead parameter $\tau_{s}$ in our numerical study in Section 4). When the number of hops is high, this accumulated overhead can consume a significant fraction of a beam-hopping window, which is typically in the order of tens of milliseconds [38,39]. Balancing these competing effects is therefore essential for efficient resource utilization and high service efficiency.

Our core contribution in this paper is the design and analysis of a low-complexity clustering algorithm that achieves this balance while offering strong empirical performance in realistic deployment scenarios. The technical novelty lies in the solution framework: a greedy disk-cover initialization to obtain feasible clusters, a projection characterization of the constrained center update, its efficient implementation via the Boyle–Dykstra method, and a convergent alternating refinement procedure. While existing cluster-based beam placement optimization approaches yield performance gains over fixed beam layouts, they do not explicitly capture the trade-off between the number of formed clusters and cluster compactness within a unified framework, nor do they provide provable convergence guarantees for balancing these competing objectives.

We propose a new geometric user clustering algorithm, called *GeoClust*, that runs in polynomial time and balances the number of clusters against cluster compactness. Its performance relative to representative benchmark schemes is quantified in Section 4. Throughout the paper, the term GeoClust refers specifically to our clustering algorithm that determines user groups and cluster centers (i.e., beam-pointing locations) for adaptive multibeam GEO satellite networks, rather than to the broader scheduling or resource-allocation framework.

The algorithm begins with a greedy disk-cover step, placing fixed-radius disks over the densest user regions to yield an initial set of feasible clusters that satisfy a prescribed maximum radius constraint. Each cluster center is then refined via a *projection-based* update, in which the arithmetic mean of the cluster users is projected onto the feasible region to ensure strict radius compliance (Theorem 1). It proceeds through alternating refinement, where cluster members and centers are iteratively updated. This alternating optimization procedure is provably convergent in a finite number of steps (Theorem 2). Finally, hyperparameter fine-tuning over the cluster radius can be performed via a one-dimensional line search, identifying a Pareto-optimal trade-off point without requiring exhaustive enumeration of cluster layouts.

Our main contributions are summarized as follows:

- We propose a polynomial-time constrained clustering algorithm (GeoClust) for GEO satellite networks that combines greedy disk-cover initialization with Boyle–Dykstra projection-based center updates to enforce cluster-radius constraints within an alternating optimization framework, while balancing cluster compactness and beam-hopping overhead.
- On a real-world population dataset, GeoClust delivers more than a twofold increase in both zero outage and median user data rates under heavy traffic conditions, outperforming all benchmark schemes considered in this paper.
- The algorithm’s aggregate downlink throughput scales almost linearly with the number of RF chains, whereas the benchmarks plateau early due to RF chain idling or high beam-switching overhead.
- GeoClust maintains its performance advantage under lighter traffic conditions (20% user activity), lifting the entire throughput distribution. It achieves over 1.8× gain in the 5th percentile and 5× gain in the 95th percentile user rates compared to the best benchmark, significantly enhancing both worst-case and best-case service levels.

These contributions offer important design insights for next-generation GEO multibeam satellite systems. They show that, under the considered beam-hopping and switching-overhead constraints, balanced beam geometry, rather than sheer beam count, is the key to improving system capacity. Geometry-aware user clustering enables highly efficient exploitation of onboard RF resources, under realistic switching delays and uneven traffic. This leads to significant gains in typical user rates, near-linear aggregate throughput scaling with RF chain count, and markedly improved per-user throughput levels. These observations underscore the importance of embedding spatial structure and hop-aware trade-offs into future GEO scheduling and beam placement frameworks.

The remainder of the paper is organized as follows. Section 2 outlines the system model for GEO satellites equipped with uniform planar array (UPA) antennas and formulates the associated user clustering optimization problem. Section 3 introduces the GeoClust algorithm and establishes its convergence and feasibility preservation properties. Section 4 presents numerical results and performance analysis of GeoClust under various traffic conditions. Finally, Section 5 concludes the paper. Technical proofs are provided in Appendix A and Appendix B.

**Scope of This Work:** This paper addresses the network-level problem of user clustering and cluster center selection for beam-pointing under a fixed nominal beam footprint. The optimization of element-level beam synthesis parameters, such as beamwidth control, null placement, and excitation design, constitutes a complementary antenna-level problem and is outside the scope of this work. This separation enables a tractable formulation of the clustering problem and allows the development of a polynomial-time algorithm with provable performance characteristics.

## 2. System Model and Problem Formulation

This section presents the system model and key parameters for optimizing ground user clustering in multibeam GEO satellite networks. It begins by describing the satellite configuration and ground user distribution.

### 2.1. Satellite Configuration and Ground User Distribution

**Figure 1:**
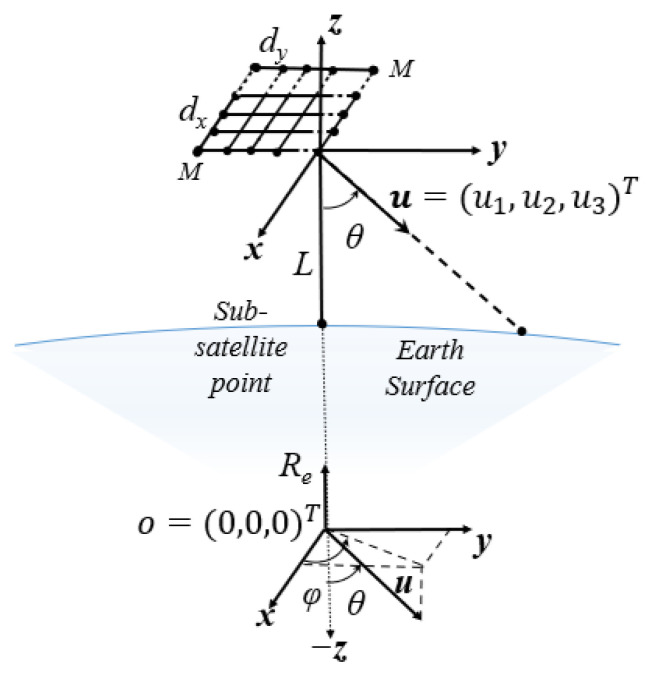
System model of GEO satellite using UPA.

Consider the downlink of a multibeam GEO satellite equipped with a square uniform planar array (UPA) antenna to serve ground users that are non-uniformly distributed over the Earth’s surface. Without loss of generality, the Earth center is defined as the origin, and the satellite position is along the *z*-axis at $s={\left(0,0,L+R_{e}\right)}^{⊤}$, where $R_{e}=6371$ [km] is the Earth radius and L= 35,786 [km] denotes the satellite’s altitude (see Figure 1).

The satellite has *K* RF chains, enabling the formation of up to *K* spot beams for spatial multiplexing of ground users. Ground users are indexed by $i\in \left[1:N\right]$, where the notation $\left[a:b\right]$ is used to denote the interval of integers from *a* to *b*. The location of ground user $i\in \left[1:N\right]$ is given by the vector $u_{i}={\left(x_{i},y_{i},R_{e}\right)}^{⊤}\in R^{3}$ and every ground user has an elevation–azimuth angle pair $\left(\theta_{i},\phi_{i}\right)$ with the satellite. There is no specific distribution imposed on the ground user location set $U=\left\{u_{1},\ldots ,u_{N}\right\}$. These locations serve as input features for the clustering problem to determine user clusters. In real-world scenarios, user distributions are typically non-uniform, with higher concentrations in urban areas and sparse populations in rural regions.

The normalized downlink channel between the satellite’s UPA antenna and the simultaneously served ground users in a given beam-hopping group is represented by a square matrix $H={\left[h_{i,j}\right]}_{i,j=1}^{K^{\prime }}\in C^{K^{\prime }\times K^{\prime }}$ [34,35], where $K^{\prime }\leq K$ is the number of the simultaneously served ground users. This matrix captures both the direct beam gains along the diagonal (i.e., intended signal paths) and the IBI through the off-diagonal elements, which is influenced by the beam pattern, array geometry, and angular separation between beam directions and user locations. Each entry $h_{i,j}$ represents the complex channel gain from the *j*th transmit beam to the *i*th ground user. Here, the *i*th user belongs to cluster $C_{j^{\prime }}$, with $j^{\prime }\neq j$, i.e., the beam–user pair corresponds to inter-cluster interference. The channel gain is given by

(1) $$\begin{array}{cc} h_{i,j}\; & =\frac{1}{M^{2}}e^{-ȷ\frac{M-1}{2}\left(\psi_{x}+\psi_{y}\right)}\frac{\sin\left(\frac{M\psi_{x}}{2}\right)}{\sin\left(\frac{\psi_{x}}{2}\right)}\frac{\sin\left(\frac{M\psi_{y}}{2}\right)}{\sin\left(\frac{\psi_{y}}{2}\right)}, \end{array}$$

where $\psi_{x}=\pi \left(\sin\theta_{j}\cos\phi_{j}-\sin\theta_{i}\cos\phi_{i}\right)$ and $\psi_{y}=\pi \left(\sin\theta_{j}\sin\phi_{j}-\sin\theta_{i}\sin\phi_{i}\right)$.

The equation indicates that each ground user located at angular coordinates $\left(\theta_{i},\phi_{i}\right)$ can receive a portion of the power transmitted by a beam steered towards a point on the Earth’s surface given by $\left(\theta_{j},\phi_{j}\right)$, accounting for the Earth’s curvature. This spatial overlap in beam radiation patterns leads to IBI, as users may receive unintended signal energy from neighboring beams.

**Remark:** We emphasize that (1) is used as a normalized array-response gain and inter-beam coupling model under a fixed UPA configuration. It is not intended as an optimization model for element-level beam synthesis. Throughout the paper, the nominal physical beam footprint radius $\rho_{b}$ is determined by the antenna configuration, while the optimization is performed only over the cluster geometry, including cluster centers and radius.

### 2.2. User Clustering Problem

The ground user clustering problem is introduced in this subsection. The main objective of optimizing the user clusters is to maximize the offered throughput by seeking a balance between having many small clusters, which forces excessive beam hopping and associated overhead, and having too few clusters, which forces large, power-inefficient clusters. In the GEO satellite downlink, every additional cluster requires an extra RF chain resource and hence increases the number of beam hops, incurring extra switching, guard, and signal processing times [1,2,34,35]. On the other hand, clustering users into fewer but larger clusters degrades the downlink performance due to higher power losses and inefficient use of the available RF chains [34].

We develop a new optimization problem that balances the number and size of clusters to optimize resource utilization while accounting for beam-hopping overhead. First, we define the user clustering rule as follows.

**Definition** **1.** *A user clustering rule $f:\left[1:N\right]\mapsto \left[1:N\right]$ is a mapping from $\left[1:N\right]$ to $\left[1:N\right]$ such that its image $f\left(\left[1:N\right]\right)$ forms an integer interval $\left[1:f_{\max}\right]$ for some $f_{\max}\in \left[1:N\right]$.*

A clustering rule *f* assigns a *unique* cluster label (indexed from 1 to $f_{\max}$) to each ground user (indexed from 1 to *N*). The parameter $f_{\max}$ represents the number of nonempty clusters (i.e., distinct cluster labels) generated by the clustering rule *f*. Since each user must belong to one cluster and each label in $\left[1:f_{\max}\right]$ is used by at least one user, the total number of clusters cannot exceed the total number of users. The set of all possible clustering rules is represented by F.

Intuitively, each user cluster should consist of geographically close users spread over an area ranging from tens to a few hundred square kilometers, such as a small town or village. Within each cluster, the Earth’s surface is approximated as a locally flat manifold and ground users access the service using time division multiple access (TDMA). This planar approximation is used only for cluster formation and center updates. It is appropriate because the clusters are geographically compact and their radius is bound by the nominal beam footprint. The impact of Earth curvature is retained in the system model through the elevation–azimuth-based channel and interference formulation in (1), ensuring that system-level performance is evaluated using the full spherical geometry.

The optimum ground user clustering problem involves jointly determining the optimum clustering rule and the cluster centers (i.e., beam-pointing locations). We formulate this joint optimization problem as follows:

(2) $$\begin{array}{c} \begin{array}{c} \begin{array}{cc} \underset{\underset{{\left\{\mu_{i}\right\}}_{i=1}^{f_{\max}}\subset P}{f\in F,}}{\mathrm{minimize}}\; & \beta \cdot \sum_{i=1}^{f_{\max}}\sum_{j\in C_{i}}{∥u_{j}-\mu_{i}∥}^{2}+\alpha \cdot g\left(f_{\max}\right)\\ \mathrm{subject}\;\mathrm{to}\; & C_{i}=\left\{j\in \left[1:N\right]:f\left(j\right)=i\right\},\forall i\in \left[1:f_{\max}\right]\\ \; & ∥u_{j}-\mu_{i}∥\leq \rho ,\forall i\in \left[1:f_{\max}\right]\;\mathrm{and}\;\forall j\in C_{i} \end{array}, \end{array} \end{array}$$

where $C_{i}$ is cluster *i* (i.e., the set of ground users with cluster label *i*), $\mu_{i}$ is the center of $C_{i}$, $P≜\left\{{\left(x,y,z\right)}^{⊤}\in R^{3}:z=R_{e}\right\}$ is the planar ground-coordinate representation used for clustering, and $∥\cdot ∥$ denotes the Euclidean norm.

The coefficient β normalizes the squared-distance term and is taken as the reciprocal of the square of the nominal beam diameter, thereby rendering the first term dimensionless. The coefficient α is a dimensionless trade-off parameter, and throughout this paper, we set α=1 after this normalization.

The function *g* is a non-decreasing cost of the number of clusters and models the clustering-layer penalty associated with beam hopping. In particular, $g\left(f_{\max}\right)$ reflects the minimum number of hops required to serve $f_{\max}$ clusters with *K* RF chains. The physical beam-hopping timing parameters, such as the hopping window duration and the switching overhead, are incorporated later in the system-level evaluation in Section 4.4.

This formulation ensures that each cluster is sufficiently compact (via the radius constraint) and that increasing the number of clusters incurs additional cost through *g*.

In the remainder of the paper, we set

(3) $$g\left(f_{\max}\right)=\left\lceil \frac{f_{\max}}{K}\right\rceil ,$$

which represents the minimum number of hops required to serve $f_{\max}$ clusters using *K* RF chains. With *K* RF chains onboard, the satellite can serve *K* clusters of users simultaneously, using *K* dynamically formed spot beams.

**Remark:** Heterogeneous traffic demand across user clusters is incorporated at the scheduling stage, in conjunction with the geometry-driven clustering optimization in (2). Specifically, once clusters are formed, they are grouped into beam-hopping sets and allocated transmission time based on their aggregate demand levels, as detailed in Section 4.4. This separation enables the clustering algorithm to operate at the *spatial* layer, while traffic variations are handled through time resource allocation across clusters at faster timescales.

Within each cluster, users are served by TDMA. Hence, the main role of the compactness term in (2) is to limit the degradation in desired gain due to user offset from the cluster center under the fixed beam model, while the second term captures the beam-hopping overhead induced by excessive cluster count.

The optimization problem in (2) is an NP-hard problem [40] since it requires simultaneously determining the optimum number of clusters and optimizing the cluster centers. This clustering problem is designed to identify an appropriate balance between the two clustering extremes by balancing the two cost terms through the maximum allowable cluster radius. In particular, our approach here follows a common multi-objective optimization technique, using trade-off parameters to identify the Pareto-optimal boundary between competing objectives [41].

## 3. Geometric User Clustering for GEO Satellite Networks

In this section, we present an efficient polynomial-time approximation algorithm to address the optimization problem in (2) by decomposing it into manageable subproblems to achieve near-optimum performance. This begins by focusing on the fundamental task of optimizing cluster centers under a radius constraint, which serves as the foundation for our overall solution.

**Figure 2:**
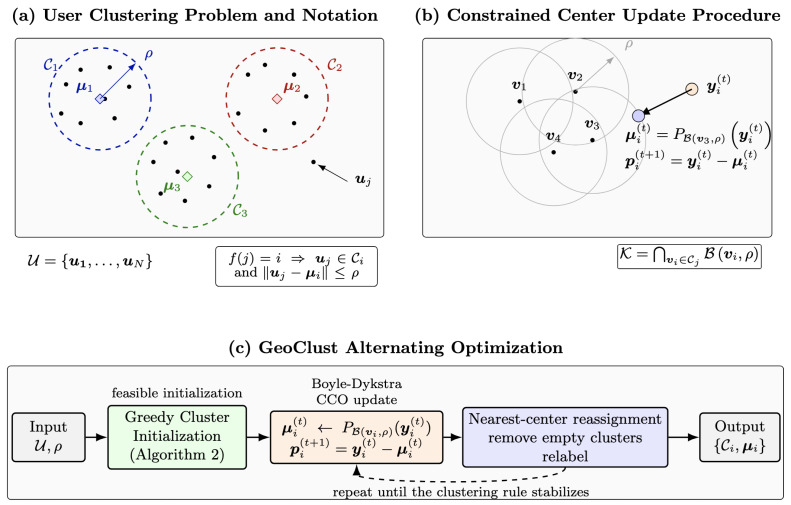
Schematic illustration of the notation and algorithmic structure of GeoClust. Panel (**a**) visualizes the user clustering rule and the radius-constrained cluster geometry used in Section 2.2. Panel (**b**) illustrates the constrained center update in Section 3.1: the arithmetic mean is projected onto the feasible set $K={⋂}_{v_{i}\in C_{j}}B\left(v_{i},\rho \right)$ to preserve the cluster-radius constraint. Panel (**c**) summarizes the alternating GeoClust procedure combining Greedy Cluster Initialization, Boyle–Dykstra-based center refinement, and nearest-center membership reassignment. The dashed circles in Panel (**a**) represent cluster-feasibility disks used to visualize the radius constraint and do not denote synthesized beam footprints.

To facilitate the presentation of the proposed method, Figure 2 provides a schematic overview of both the problem formulation and the GeoClust algorithm. The figure first visualizes the ground user set, clustering rule, and the radius-constrained cluster geometry introduced in Section 2.2. It then illustrates the key constrained center update procedure, where the cluster centroid is projected onto the intersection of feasibility disks to enforce the radius constraint. Finally, it summarizes the alternating optimization structure of GeoClust, combining greedy initialization, constrained center refinement, and membership reassignment. This schematic serves as a visual reference for the notation and algorithmic steps used in the remainder of the paper.

### 3.1. Cluster Center Optimization

Optimizing the locations of cluster centers in (2) is important because the distance between a cluster point and the cluster center (i.e., beam-pointing direction) directly impacts the achievable data rate for users accessing the service within the cluster. To analyze this problem, we first consider a generic cluster $C=\{v_{1},\ldots ,v_{n}\}$, where we represent the cluster by the user locations but not by their indices, with a slight abuse of notation. We consider that the cluster points belong to P.

**Definition** **2.** *The diameter of a set $S\subseteq R^{3}$ is defined as $\mathrm{diam}\left(S\right)≜\sup_{u,v\in S}∥u-v∥$.*

**Definition** **3.** *For a compact subset S of $R^{3}$, the projection of a point $v\in R^{3}$ on S is any point $u^{⋆}\in S$ that achieves $\min_{u\in S}∥u-v∥$.*

We will use the notation $P_{S}\left(v\right)$ to represent a projection of v onto the set S. The Cluster Center Optimization problem for cluster C is the problem of finding the optimum cluster center while satisfying the cluster-radius constraint, which is given by

(4) $$\begin{array}{c} \begin{array}{cc} \underset{\mu \in P}{\mathrm{minimize}}\; & \sum_{i=1}^{n}{∥v_{i}-\mu ∥}^{2}\\ \mathrm{subject}\;\mathrm{to}\; & ∥v_{i}-\mu ∥\leq \rho ,\forall i\in \left[1:n\right] \end{array}. \end{array}$$

The following theorem characterizes the solution for (4).

**Theorem** **1.** *The Cluster Center Optimization* (4)*, whenever it is feasible, has a unique solution given by $P_{K}\left(\bar{v}\right)$, where $\bar{v}=\frac{1}{n}\sum_{i=1}^{n}v_{i}$ is the arithmetic mean of cluster points, and *

$$K=\overset{n}{\underset{i=1}{⋂}}B\left(v_{i},\rho \right)$$

*is the intersection of the P-balls $B\left(v,\rho \right)≜\left\{\mu \in P:∥v-\mu ∥\leq \rho \right\}$.*

**Proof.**  See Appendix A.    □

Establishing the feasibility of (4) is a fundamental problem in computational geometry. In particular, (4) is feasible if and only if $r^{⋆}\leq \rho$, where $r^{⋆}$ denotes the radius of the smallest P-ball $B^{⋆}$ that encloses all points in C. Efficient linear-time algorithms exist to compute $B^{⋆}$ [42,43]. A tight upper bound on $r^{⋆}$ is given by $r^{⋆}\leq \frac{\mathrm{diam}\left(C\right)}{\sqrt{3}}$ [44]. Hence, a sufficient condition for the feasibility of (4) is $\mathrm{diam}\left(C\right)\leq \sqrt{3}\rho$. To ensure feasibility in our clustering algorithm, we will initialize all clusters such that their points are enclosed within P-balls of radius ρ. We will further elaborate on this initialization process in the next section.

Theorem 1 establishes a fundamental structural property of the solution to (4). The feasible set K is a convex compact subset of P, given by the intersection of multiple P-balls. This structure enables efficient computation of the projection onto K.

In particular, Boyle–Dykstra’s algorithm [45] provides an efficient iterative method by cyclically projecting onto the individual balls $B\left(v_{i},\rho \right)$, while maintaining auxiliary correction vectors that account for the residual introduced by previous projections.

Each projection onto a single P-ball admits a closed-form expression

(5) $$\begin{array}{c} P_{B(v_{i},\rho )}\left(y\right)=\left\{\begin{array}{cc} v_{i}+\rho \frac{y-v_{i}}{∥y-v_{i}∥}, & \;∥y-v_{i}∥>\rho \\ y, & \mathrm{otherwise} \end{array}\right., \end{array}$$

and is therefore computationally simple. As a result, the overall projection onto K can be computed efficiently through successive projections onto individual balls. This projection step is the key methodological ingredient in GeoClust, as it replaces the standard centroid update (i.e., the arithmetic mean of cluster points) with a radius-constrained center update obtained via projection, ensuring that the cluster-radius constraint is satisfied at every iteration. The algorithm for solving (4) is given in Algorithm 1.

In Algorithm 1, $p_{i}^{\left(t\right)}$ denotes the correction vector associated with the ball $B\left(v_{i},\rho \right)$ at outer iteration *t*. The intermediate point $y_{i}^{\left(t\right)}$ is the current iterate after applying this correction, $\mu_{i}^{\left(t\right)}$ is the projection of $y_{i}^{\left(t\right)}$ onto the *i*th ball. The update $p_{i}^{\left(t+1\right)}=y_{i}^{\left(t\right)}-\mu_{i}^{\left(t\right)}$ stores the residual generated by that projection and carries it forward to subsequent iterations. This standard Boyle–Dykstra correction step enables convergence to the exact projection onto K, rather than to an approximate solution obtained by simple cyclic projections.
**Algorithm 1** Cluster Center Optimization Algorithm  1:**Input:** Cluster points $\left\{v_{1},\ldots ,v_{n}\right\}\subset P$, radius ρ>0  2:**Initialize:**  $\bar{v}\leftarrow \frac{1}{n}\sum_{i=1}^{n}v_{i}$, $\mu^{\left(0\right)}\leftarrow \bar{v}$, $p_{i}^{\left(0\right)}\leftarrow 0$ for i=1,…,n, t←0  3:**repeat**                                                                                   ▹Boyle–Dykstra Iteration  4:      $\mu_{0}^{\left(t\right)}\leftarrow \mu^{\left(t\right)}$  5:      **for** i=1 to *n* **do**  6:            $y_{i}^{\left(t\right)}\leftarrow \mu_{i-1}^{\left(t\right)}+p_{i}^{\left(t\right)}$  7:            $\mu_{i}^{\left(t\right)}\leftarrow P_{B(v_{i},\rho )}\left(y_{i}^{\left(t\right)}\right)$  8:            $p_{i}^{\left(t+1\right)}\leftarrow y_{i}^{\left(t\right)}-\mu_{i}^{\left(t\right)}$  9:      **end for**10:      $\mu^{\left(t+1\right)}\leftarrow \mu_{n}^{\left(t\right)}$, t←t+111:**until** Convergence                                               ▹Stop when $∥\mu^{\left(t\right)}-\mu^{\left(t-1\right)}∥$ is small12:**Return:** μ as $P_{K}\left(\bar{v}\right)$.

Algorithm 1 establishes an efficient procedure to optimize the first term in the ground user clustering problem in (2) for a given set of clusters. In particular, it can be applied to all the clusters formed by a clustering rule *f* in (2) to optimize their centers subject to cluster-radius constraints. However, this solution does not yet address the problem of how to choose *f* to form these clusters efficiently in the first place, aiming to minimize the second term in the objective function in (2). To this end, we explain our approach for initializing the clusters in the next section, which will eventually lead to an iterative process to update cluster centers and cluster memberships.

### 3.2. Greedy Cluster Initialization

We adopt a geometric disk-cover algorithm to initialize the clusters, which is given in Algorithm 2. The algorithm iteratively selects the disk centered at a ground user location that covers the maximum number of uncovered points, continuing this process until all ground user locations are covered. This approach provides an efficient initialization for the clusters with a runtime of $O\left(N^{2}\right)$ and also yields a near-optimal cover [46,47].

An important feature of Algorithm 2 is that it covers all ground user locations with a small number of P-balls of radius ρ. This is important because the initial cluster formation must ensure that the Cluster Center Optimization problem in (4) remains feasible to develop an alternating optimization process to update clusters. Feasibility of (4) is guaranteed if the clusters $C_{1},\ldots ,C_{f_{\max}}$ are formed such that the ground user locations within each cluster $C_{i}$ can be enclosed within a P-ball of radius ρ. The initial set of clusters produced by Algorithm 2 satisfies this condition, and hence Algorithm 1 can in turn be applied to optimize cluster centers for all clusters individually.
**Algorithm 2** Greedy Cluster Initialization Algorithm  1:**Input:** Ground user locations $U=\left(u_{1},\ldots ,u_{N}\right)\subset P$, disk radius ρ  2:**Output:** Cluster centers $\left\{\mu_{1},\ldots ,\mu_{f_{\max}}\right\}$ and clusters $\left(C_{1},\ldots ,C_{f_{\max}}\right)$  3:**Initialize:** $U_{\mathrm{rem}}\leftarrow U$, $f_{\max}\leftarrow 0$  4:**for** each $u_{i}\in U$ **do**  5:      Compute the P-ball $B_{i}=\left\{u\in P:∥u-u_{i}∥\leq \rho \right\}$  6:      Set coverage count $c_{i}=\left|B_{i}\cap U\right|$  7:**end for**  8:**while** $U_{\mathrm{rem}}\neq Ø$ **do**  9:      Let $i^{*}\leftarrow \arg\max_{i\in \left[1:N\right]}\left\{c_{i}:u_{i}\in U_{\mathrm{rem}}\right\}$10:      $f_{\max}\leftarrow f_{\max}+1$11:      Set cluster center $\mu_{f_{\max}}\leftarrow u_{i^{*}}$12:      Define cluster $C_{f_{\max}}\leftarrow \left\{i\in \left[1:N\right]:u_{i}\in B_{i^{*}}\cap U_{\mathrm{rem}}\right\}$13:      Set $U_{\mathrm{rem}}\leftarrow U_{\mathrm{rem}}\setminus B_{i^{*}}\cap U_{\mathrm{rem}}$14:      Update coverage counts $c_{i}\leftarrow \left|B_{i}\cap U_{\mathrm{rem}}\right|$ for all $u_{i}\in U_{\mathrm{rem}}$15:**end while**16:**Cluster Membership Update:**17:**for** $i\in \left[1:N\right]$ **do**18:      Assign user *i* to cluster $C_{j}$ with the nearest center, i.e., minimizing $∥u_{i}-\mu_{j}∥$19:**end for**20:**Return** $\left\{\mu_{1},\ldots ,\mu_{f_{\max}}\right\}$ and $\left\{C_{1},\ldots ,C_{f_{\max}}\right\}$

In addition to the greedy cluster formation, Algorithm 2 performs a cluster membership update at the final step before returning the clusters, where each ground user is assigned a unique cluster label based on its Euclidean distance to the cluster centers. This update is also important because the greedy cluster formation does not ensure that every ground user is assigned to the closest cluster. Reassigning users in this manner does not violate the minimum disk-cover property to cover all ground users with P-balls of radius ρ. This is because if a user is reassigned to a different cluster, then the new cluster center is necessarily closer than the originally assigned center, ensuring that the user remains within the P-ball of radius ρ centered at the updated cluster center.

**Remark:** It is well known that finding a minimum number of disks to cover a given set of points is NP-hard (e.g., see [22,23,24]). Several polynomial-time approximation algorithms have been developed to solve the problem for a set of discrete points in Euclidean space [24,48,49]. In this paper, we choose to adopt the greedy approach in Algorithm 2 due to its runtime and near-optimal cover properties.

### 3.3. GeoClust: Geometric User Clustering Algorithm for GEO Satellite Networks

In this section, we present our final clustering algorithm, termed the GeoClust algorithm, which is tailored for ground user clustering in GEO satellite networks. The GeoClust algorithm is given in Algorithm 3.

GeoClust is an alternating optimization algorithm where cluster memberships and centers are updated sequentially, refining the clustering structure at each step. It is built upon two core components introduced in previous sections: the Greedy Cluster Initialization (GCI) algorithm and the Cluster Center Optimization (CCO) algorithm.

Specifically, the GCI algorithm provides an initial clustering configuration that guarantees the feasibility of the Cluster Center Optimization problem in (4) and thereby enables the cluster center updates. Subsequently, the CCO algorithm refines the cluster centers via iterative projection onto convex constraint sets using the Boyle–Dykstra procedure, as detailed in Algorithm 1. Cluster memberships are updated based on proximity to these new centers. If the reassignment step produces any empty clusters, those clusters are removed and the remaining clusters are relabeled consecutively, so that $f_{\max}$ always equals the number of nonempty clusters. This alternating optimization process, reminiscent of the *K*-means iteration, is repeated until the clustering rule no longer changes.

We note that, unlike a simple arithmetic mean update, the CCO update ensures that the radius constraints in (2) are satisfied at each step of the algorithm, ensuring all clusters remain feasible throughout the alternating optimization and refinement process. The distinguishing feature of GeoClust, relative to unconstrained centroid-based updates, is the Boyle–Dykstra-based constrained center update, which maintains the radius constraint throughout the alternating iteration.
**Algorithm 3** GeoClust: Iterative Geometric User Clustering Algorithm for GEO Satellite Networks  1:**Input:** Ground user locations $U=\left\{u_{1},\ldots ,u_{N}\right\}\subset P$, disk radius ρ  2:**Output:** Final cluster centers $\left\{\mu_{1},\ldots ,\mu_{f_{\max}}\right\}$ and clusters $\left\{C_{1},\ldots ,C_{f_{\max}}\right\}$  3:**Initialization:**  4:    Run the GCI algorithm to obtain initial clusters $\left\{C_{1}^{\left(0\right)},\ldots ,C_{f_{\max}}^{\left(0\right)}\right\}$ and initial centers $\left\{\mu_{1}^{\left(0\right)},\ldots ,\mu_{f_{\max}}^{\left(0\right)}\right\}$  5:    Set t←0  6:**repeat**  7:      **for** i=1 to $f_{\max}$ **do**  8:            Compute the arithmetic mean of cluster *i*:$${\bar{u}}_{i}^{\left(t\right)}\leftarrow \frac{1}{|C_{i}^{\left(t\right)}|}\sum_{j\in C_{i}^{\left(t\right)}}u_{j}$$  9:            Define the feasibility set for cluster *i*:$$K_{i}=\underset{j\in C_{i}^{\left(t\right)}}{⋂}B\left(u_{j},\rho \right)$$10:            Update the cluster center using the CCO Alg:$$\mu_{i}^{\left(t+1\right)}\leftarrow P_{K_{i}}\left({\bar{u}}_{i}^{\left(t\right)}\right)$$11:      **end for**12:      **for** each ground user $u_{i}\in U$ **do**13:            Reassign $u_{i}$ to the cluster with the nearest center:$$i\in C_{j}^{\left(t+1\right)}\;\mathrm{where}\;j=\arg\min_{k}∥u_{i}-\mu_{k}^{\left(t+1\right)}∥$$                                                                   ▹In case of ties, retain the current cluster label14:      **end for**15:      Remove empty clusters and relabel the remaining clusters consecutively16:      Set $f_{\max}\leftarrow \left|\left\{j:C_{j}^{\left(t+1\right)}\neq \emptyset \right\}\right|$17:      t←t+118:**until** Convergence                             ▹Stop when the clustering rule no longer changes19:**Return:** $\left\{\mu_{1}^{\left(t\right)},\ldots ,\mu_{f_{\max}}^{\left(t\right)}\right\}$ and $\left\{C_{1}^{\left(t\right)},\ldots ,C_{f_{\max}}^{\left(t\right)}\right\}$

We provide key structural properties of the GeoClust algorithm in Theorem 2 below. In particular, we show that the algorithm is guaranteed to converge and that the Cluster Center Optimization subproblem remains feasible throughout the iterative process. These properties follow from the construction of the initial clusters via the GCI algorithm and the projection-based updates performed by the CCO Algorithm.

**Theorem** **2.** *The GeoClust algorithm (Algorithm 3) satisfies the following properties:*

*1*. ***Convergence:** The sequence of clustering rules generated by the GeoClust algorithm stabilizes after finitely many outer iterations.*
*2*. ***Feasibility Preservation:** At every iteration t, for each cluster $C_{i}^{\left(t\right)}$, the corresponding Cluster Center Optimization subproblem in* (4) *is feasible, i.e., there exists a point $\tilde{\mu_{i}}\in P$ such that the ground user locations of users that belong to $C_{i}^{\left(t\right)}$ are within a distance of ρ from $\tilde{\mu_{i}}$.*

**Proof.**  See Appendix B. □

### 3.4. Selection of Optimum Cluster Radius

The cluster radius ρ is a critical parameter in our GeoClust algorithm. It directly influences the overall cost function in (2) for any trade-off parameter α>0. Specifically, setting ρ too small leads to an excessive number of clusters (akin to overfitting), thereby increasing the beam-hopping overhead captured by the term $g\left(f_{\max}\right)$ in (2). On the other hand, a large value of ρ produces too few, too large clusters (akin to underfitting), resulting in higher intra-cluster distances, and thereby a larger value of the sum-distance term in the cost function. Hence, there exists an optimum cluster radius, denoted by $\rho^{⋆}$, that lies within the allowable range $\left[\rho_{\min},\rho_{\max}\right]$ and minimizes the overall cost.

This optimal $\rho^{⋆}$ can be viewed as a hyperparameter that must be learned from the data. Our proposed ground user clustering framework transforms the problem of learning $\rho^{⋆}$ into a one-dimensional line search, even though there is no closed-form expression for $\rho^{⋆}$ due to the problem complexity. This is analogous to hyperparameter tuning in the machine learning context. For each candidate radius $\rho \in \left[\rho_{\min},\rho_{\max}\right]$, the GeoClust algorithm is run in polynomial time to evaluate the corresponding cost. The optimal radius is then selected as the value that minimizes the cost function to balance the trade-off between the two competing cost terms.

Specifically, the search interval is discretized using a sufficiently fine step size $\Delta_{\rho }$. For each candidate radius ρ, the GeoClust algorithm is run to convergence and the resulting objective value in (2) is recorded. The selected radius is then

(6) $$\begin{array}{c} \rho^{⋆}=\underset{\rho \in R_{\rho }}{\arg\min}\;J^{⋆}\left(\rho \right), \end{array}$$

where $R_{\rho }$ denotes the discretized search grid and $J^{⋆}\left(\rho \right)$ is the converged cost at radius ρ. This reduces radius selection to a transparent one-dimensional search, without exhaustive enumeration of cluster layouts.

The upper bound $\rho_{\max}$ on the cluster radius can be set equal to the beam radius, which is determined by the configuration and number of antenna elements onboard the GEO satellite. This reflects the maximum coverage area of a single beam. On the other hand, the lower bound $\rho_{\min}$ is determined by the population distribution and urban density, capturing the smallest geographical area (e.g., a village or neighborhood) within which the ground users should be served together by a single beam. We will consider $\rho_{\min}$ as a given parameter in our framework, based on domain-specific knowledge regarding the expected minimum service area (e.g., the size of a typical village or neighborhood). Alternatively, the modified density-based methods [50] may be employed to estimate an appropriate value for $\rho_{\min}$ if such domain knowledge is unavailable.

## 4. Numerical Results

### 4.1. Simulation Setup

A multibeam GEO satellite equipped with a 252×252 UPA antenna, with physical dimensions of 1.68 m ×1.68 m, is considered to provide service to ground users. Operating at 20 GHz with half-wavelength antenna spacing, the UPA generates multiple spot beams, each covering a footprint with radius of $\rho_{b}=125$ km at GEO altitude.

The satellite payload includes *K* RF chains, where $K\in \left\{4,8,16,32\right\}$ is treated as a design parameter. Once user clusters are formed, they are served in a round-robin manner, with each group consisting of at most *K* clusters. This corresponds to the beam-hopping mode of operation, in which only a subset of clusters is illuminated during each transmission frame.

We take the beam-hopping window in our simulations to be $T_{H}=50$ ms as a representative scheduler-level service interval for the considered GEO beam-hopping system. This choice is motivated by the 3rd Generation Partnership Project (3GPP) service and QoS timescales that are relevant to integrated 5G/6G satellite access, including 10–50 ms transfer-interval targets in TS 22.104 and 50 ms packet delay budgets in TS 23.501 [51,52,53]. This choice fixes the common beam-hopping service timescale in the simulations, so all schemes are compared under the same latency requirement. We also consider hybrid beamforming with regularized zero-forcing (RZF) in the digital domain to mitigate inter-beam interference between user clusters scheduled for simultaneous illumination [54]. Table 1 provides system-level simulation parameters used throughout this section.

### 4.2. Population Dataset and Preprocessing

Our simulations use a real geospatial dataset of non-uniform user distribution. This dataset is extracted from the Australian population grid for 2023 provided by the Australian Bureau of Statistics (ABS) [55]. The ground users in this dataset span a geographical region of 4000 km × 4000 km. Each data point in this dataset represents an area of one square km expressed as a pixel and is given as a triplet ($x_{i},y_{i},\zeta_{i}$), where $\zeta_{i}$ is the population density in one pixel centered at $\left(x_{i},y_{i}\right)$ referenced to the south-west corner of the map.

**Figure 3:**
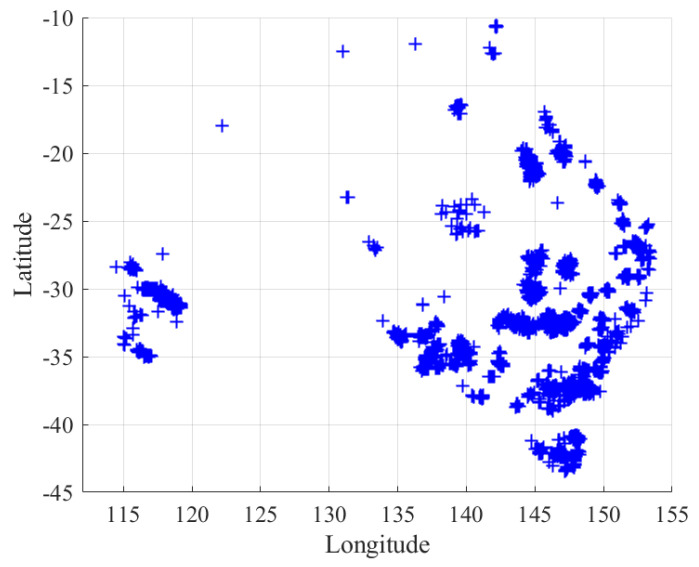
Geographical distribution of ground users in the simulation dataset extracted from the real-world Australian population data [55].

The dataset is preprocessed to focus on regional Australia, where the GEO satellite service is most in demand. Specifically, the population density value $\zeta_{i}$ for each grid cell is clipped to the range $\left[0,\zeta_{th}\right]$, where $\zeta_{th}=1.13$ persons per square kilometer, based on the 2023 estimated resident population density for regional Australia [56]. The preprocessed dataset contains 7570 data points, representing ground users distributed over regional Australia (see Figure 3). The user distribution is highly non-uniform and exhibits clustering, reflecting the tendency of populations in remote areas to concentrate around towns, service centers, or transport corridors.

Unless otherwise stated, each spatial dataset used in this section is treated as a fixed realization. Accordingly, for a fixed dataset, activity pattern, and system configuration, the outage curves and the median and percentile throughput values reported below are empirical spatial statistics over the users in that dataset, rather than Monte Carlo averages over repeated user location realizations.

### 4.3. Example Operation of GeoClust Algorithm

**Figure 4:**
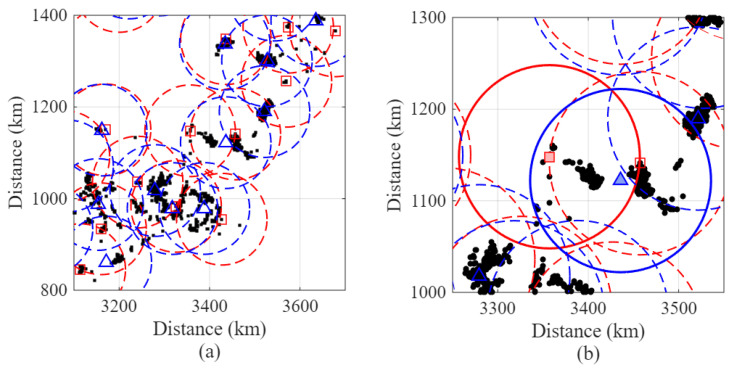
Example operation of the GeoClust algorithm in (**a**) overall view and (**b**) one-cluster view. Dashed circles denote GeoClust cluster-feasibility disks of radius ρ. They are not synthesized antenna radiation patterns or beam footprints.

We first present an illustrative example to demonstrate the operation of the GeoClust algorithm. The example focuses on a subsection of the dataset shown in Figure 3, specifically the region spanning longitudes 3100 km to 3700 km and latitudes 800 km to 1400 km (see Figure 4a). The ground users are marked by black dots in the figure.

The GeoClust algorithm first initializes the ground user clusters (for a given cluster radius) using the GCI algorithm in Algorithm 2. The red square symbols in Figure 4a show the initial locations of the cluster centers, and the dotted red circles show the boundaries of these clusters. These circles represent the cluster-feasibility regions defined by the radius constraint in (2) and are used for visualization of the clustering process. They do not correspond to synthesized antenna beam patterns or variable beamwidth designs.

Following this step, the CCO algorithm in Algorithm 1 refines the cluster centers, and cluster memberships are updated based on proximity to the new centers. The new locations of cluster centers are marked using blue triangle symbols in Figure 4a, and the dotted blue circles are the boundaries of the clusters after updating the cluster centers at convergence. To provide better resolution, a cluster from Figure 4a is zoomed in and shown in Figure 4b, with the selected cluster highlighted using solid lines. The cluster radius used in this figure is ρ=100 km. We note that this radius is a clustering parameter that governs user grouping and does not imply adaptive beamwidth control.

**Figure 5:**
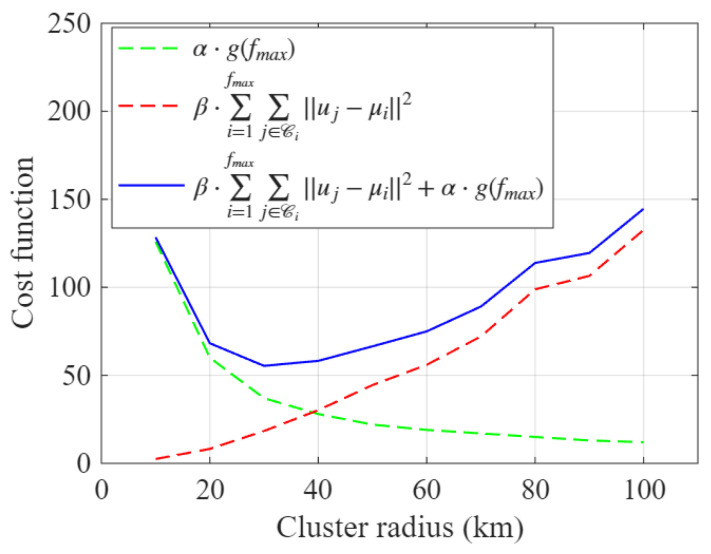
The compactness term, beam-hopping overhead term, and total objective evaluated over the discrete radius search grid for K=8.

Figure 5 presents a numerical illustration of cluster-radius optimization within the GeoClust framework for K=8. As discussed earlier, the cluster radius ρ is treated as a tunable hyperparameter. To determine the optimum value, the interval $\left[\rho_{\min},\rho_{\max}\right]$ is discretized into a finite grid. For each candidate radius, GeoClust is run to convergence, and the corresponding values of the cost function in (2) are evaluated. The optimal operating point is then selected as the grid point that minimizes the total cost, which is formally given by (6).

The figure separately plots the two constituent terms of the cost function, representing spatial compactness and beam-hopping overhead, as well as their sum, for varying values of ρ with K=8. As observed, the two cost terms exhibit opposing trends: increasing ρ reduces beam-hopping overhead but increases intra-cluster distances. Their sum forms a convex-looking curve, with a unique minimum. The cluster radius that minimizes this combined cost is selected as the optimum operating point. In this example, the optimum cluster radius is $\rho^{⋆}=30$ km.

### 4.4. User Outage and Throughput Performance

To evaluate user outage and throughput performance, we compare the GeoClust algorithm against the following benchmark beam placement approaches:

- *Fixed-Beam Distribution Beam Placement (FBD-BP):* Spot beams are directed toward a set of predefined beam locations, independent of user distribution. This baseline uses the nominal beam footprint radius $\rho_{b}$ by construction.
- *Low-Complexity User Density-Based Beam Placement Design (LCUD-BPD):* This is a geometric beam-placement baseline method introduced in [28] that utilizes user density to guide beam placement decisions with low overhead. The method operates with variable cluster radii subject to prescribed admissible radius lower and upper bounds. In our implementation, these bounds are set to 0 and $\rho_{b}$. The LCUD-BPD algorithm is orbit-agnostic in design, depending solely on ground user coordinates and tunable radius constraints (lower and upper bounds), without incorporating satellite-specific kinematic parameters. In our GEO satellite setting, the admissible radius interval is set to $\left[0,\rho_{b}\right]$.
- *Per-User Beam Placement (PU-BP):* Each beam is individually pointed at a single ground user, resulting in *N* clusters, each containing one ground user. This scheme bypasses the clustering step and serves as a user-centric extreme reference, representing the finest possible beam-pointing granularity under the adopted fixed-footprint model. It is included to expose the opposite end of the design space, where the gain from per-user beam pointing is offset by the resulting increase in the number of beam hops and associated overhead.

**Remark 1:** Our benchmark set is selected to represent three distinct reference points for the GEO beam-placement problem: FBD-BP as a fixed-footprint baseline, LCUD-BPD as a variable-radius geometric placement baseline, and PU-BP as a user-centric no-clustering extreme. Standard *K*-means is not included as a primary benchmark because it requires the number of clusters to be specified a priori and does not enforce the hard cluster-radius constraint in (2). In contrast, the present formulation treats both the number of clusters and radius feasibility as intrinsic design variables. A fair *K*-means-based comparator would therefore require an outer search over the number of clusters, together with post-processing to enforce feasibility, and would no longer represent standard *K*-means.

**Remark 2:** The PU-BP scheme is included as a diagnostic reference rather than as a practical beam-hopping design target. Under the common scheduling and resource-allocation framework used for all schemes, it isolates the effect of extreme beam-pointing granularity. In particular, if the per-hop overhead is negligible, one-beam-per-user service becomes attractive, whereas for realistic nonzero overheads the resulting number of beam hops becomes the dominant bottleneck. This makes PU-BP useful for interpreting the numerical results for different per-hop overhead regimes, and for locating GeoClust relative to both practical clustering baselines and the user-centric no-clustering extreme.

To enable throughput and outage performance analysis, both GeoClust and the benchmark beam placement algorithms are integrated with beam scheduling and resource allocation procedures. Beam scheduling is required because clustering alone does not determine which beams are activated when and how resources are allocated across users. To this end, the beam scheduling in our simulations is performed using Algorithm 2 in [31], which mitigates IBI through distance-based spatial reuse.

We consider full frequency reuse across simultaneously illuminated beams. IBI is managed through the adopted beam-hopping scheduler and RZF precoding framework. We adopt a fixed power resource allocation scheme for fairness and simplicity, where each spot beam is assigned the same transmit power for transmissions.

The clustering stage determines the spatial grouping of users, while the scheduling stage incorporates traffic heterogeneity across clusters through time resource allocation. In particular, a fractional slot allocation scheme is employed by forming beam scheduling groups $G_{1},\ldots ,G_{L}$. Each $G_{l}$ represents a set of clusters that are simultaneously illuminated during a beam-hopping time slot. Every $G_{l}$ is allocated a time slot $\lambda_{l}$ as a fraction of the overall beam-hopping window $T_{H}$ according to

(7) $$\lambda_{l}=\frac{\max_{C\in G_{l}}\;\Omega_{C}}{\sum_{l=1}^{L}\max_{C\in G_{l}}\;\Omega_{C}}T_{H},$$

where $\Omega_{C}$ is the demand level of a cluster C. The cluster demand levels are calculated by considering the number of ground users in each cluster and the requested data rates per user.

The allocated time for each cluster group accounts for both the transmission duration and a per-hop non-data overhead. In our simulations, this overhead is represented by the effective parameter $\tau_{s}$, which aggregates beam switching time, guard time, and related control and processing delay into a single value. For brevity, we refer to $\tau_{s}$ as the beam-switching overhead below. Following the DVB-S2X beam-hopping waveform and superframe timing guidelines, which include beam transition intervals, guard symbols, and superframe signaling overhead, we consider two representative overhead values: $\tau_{s}=50$μs and $\tau_{s}=20$μs [57,58]. In addition, an idealized case with $\tau_{s}=0$ is included to assess the performance upper bound in the absence of per-hop overhead. This fixed $\tau_{s}$ abstraction provides an effective system-level representation of per-hop overhead, capturing the aggregate impact of switching, guard, and control delays while maintaining a tractable and implementation-agnostic model.

#### 4.4.1. User Outage Performance

We first present the outage performance results for our proposed GeoClust algorithm as well as for the benchmark schemes. To this end, we define the offered data rate per ground user according to

(8) $$R_{i}=\frac{T_{i}^{\mathrm{tx}}}{T_{H}}B\log_{2}\left(1+\gamma_{i}\right),$$

where *B* is the total bandwidth, $T_{i}^{\mathrm{tx}}$ is the user allocated time for downlink transmission and $\gamma_{i}$ is the SINR at the user location. This effective-rate metric captures both physical-layer link quality through $\gamma_{i}$, and the service delay induced by beam hopping and TDMA time sharing through $\frac{T_{i}^{\mathrm{tx}}}{T_{H}}$ since $R_{i}$ is normalized by the beam-hopping window $T_{H}$. The user transmission times are computed by considering beam-hopping time fractions allocated to cluster groups and using TDMA for intra-cluster time resource allocation.

The SINR values in (8) are computed as follows, following similar steps given in [34,35]. Consider a beam-hopping group $G_{l}=\left\{C_{1,l},\ldots ,C_{m_{l},l}\right\}$ with $m_{l}=\left|G_{l}\right|\leq K$, and let $\mu_{j,l}$ denote the center of cluster $C_{j,l}$. During a given intra-cluster TDMA instant within the slot allocated to $G_{l}$, one ground user is scheduled from each active cluster. Denoting the scheduled user from $C_{j,l}$ by $u_{j,l}\in C_{j,l}$, the effective normalized channel matrix for that instant is $H_{l}={\left[h_{p,q}^{\left(l\right)}\right]}_{p,q=1}^{m_{l}}$, where $h_{p,q}^{\left(l\right)}$ is obtained from (1) by evaluating the array-response gain at the angular coordinates of $u_{p,l}$ while steering the *q*th beam toward the cluster center $\mu_{q,l}$. Equivalently, in (1), $\left(\theta_{i},\phi_{i}\right)$ is replaced by the angular pair of the scheduled user $u_{p,l}$ and $\left(\theta_{j},\phi_{j}\right)$ by the steering angles of the cluster center $\mu_{q,l}$. Hence, the diagonal entries of $H_{l}$ represent the desired beam gains seen by the scheduled users, whereas the off-diagonal entries capture the inter-beam interference generated by the other simultaneously active beams.

Let $D_{l}=\left[d_{1,l},\ldots ,d_{m_{l},l}\right]$ denote the corresponding RZF digital precoder, and let $h_{p,l}^{⊤}$ be the *p*th row of $H_{l}$. The instantaneous SINR of the scheduled user $u_{p,l}$ is then given by

(9) $$\begin{array}{c} \gamma_{p,l}=\frac{|h_{p,l}^{⊤}d_{p,l}{|}^{2}}{{\mathrm{SNR}}^{-1}+\sum_{\begin{array}{c} q=1\\ q\neq p \end{array}}^{m_{l}}{\left|h_{p,l}^{⊤}d_{q,l}\right|}^{2}},\;\mathrm{SNR}=\frac{GP}{\sigma^{2}}, \end{array}$$

where *P* is the transmit power per active beam, *G* is the antenna array gain, and $\sigma^{2}$ is the receiver noise power. For a generic ground user *i*, the quantity $\gamma_{i}$ in (8) is the value of (9) obtained when that user is scheduled within its cluster. Correspondingly, $T_{i}^{\mathrm{tx}}$ in (8) denotes the net transmission time allocated to user *i* after accounting for the beam-hopping slot duration, the per-hop overhead $\tau_{s}$, and the intra-cluster TDMA sharing.

The empirical user outage probability distribution characterizes the fraction of ground users whose achieved data rates are below a target threshold. It is defined as

(10) $$F\left(R_{0}\right)≜\frac{1}{N}\sum_{i=1}^{N}1_{\left\{R_{i}<R_{0}\right\}},$$

where $R_{0}$ is the requested rate and $R_{i}$ is the data rate achieved by the *i*th ground user, computed according to (8). Here, $1_{\left\{\cdot \right\}}$ denotes the indicator function.

**Figure 6:**
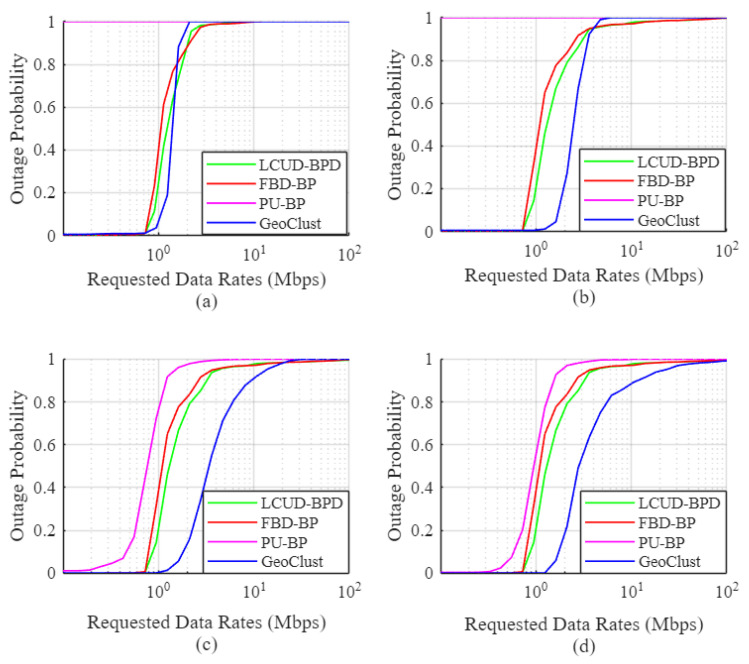
User outage for GeoClust, LCUD-BPD, FBD-BP and PU-BP for $\tau_{s}=50$μs and (**a**) K=4, (**b**) K=8, (**c**) K=16 and (**d**) K=32.

**Figure 7:**
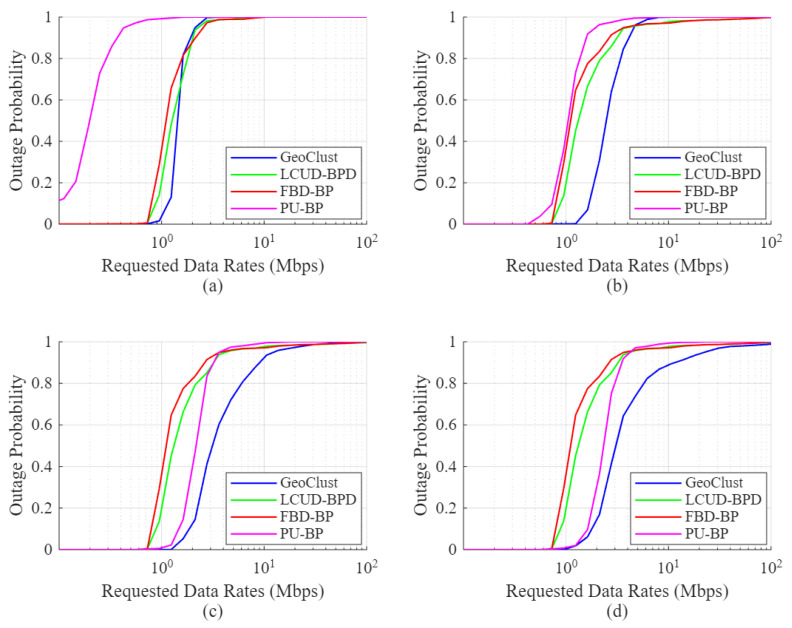
User outage for GeoClust, LCUD-BPD, FBD-BP and PU-BP for $\tau_{s}=20$μs and (**a**) K=4, (**b**) K=8, (**c**) K=16 and (**d**) K=32.

**Figure 8:**
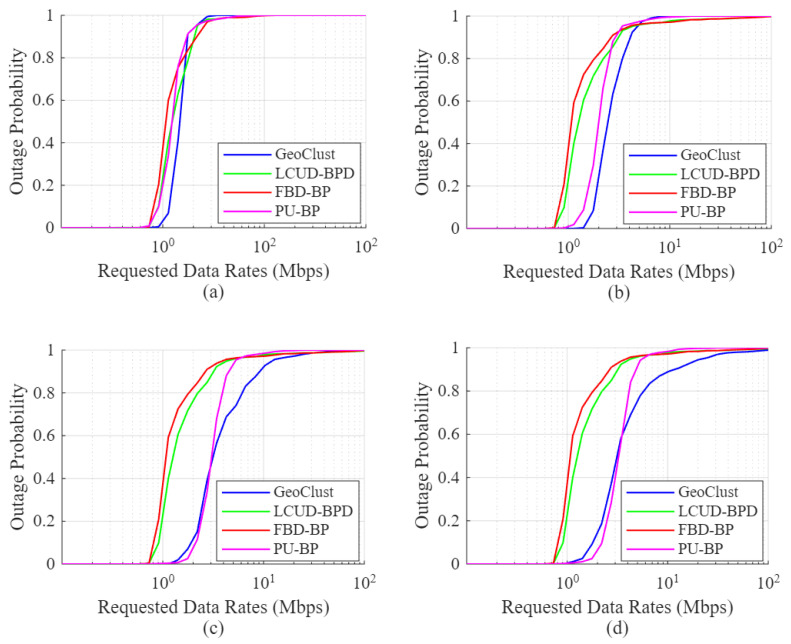
User outage for GeoClust, LCUD-BPD, FBD-BP and PU-BP without beam switching overhead ($\tau_{s}=0$) and (**a**) K=4, (**b**) K=8, (**c**) K=16 and (**d**) K=32.

Figure 6, Figure 7 and Figure 8 show the empirical user outage probability of the GeoClust algorithm, in comparison to LCUD-BPD, FBD-BP, and PU-BP, using the Australian population dataset. The evaluation is conducted under a heavily congested scenario in which all ground users are assumed to be active simultaneously. The outage probability is plotted as a function of the target data rate for varying numbers of RF chains and beam switching overheads. Both practical ($\tau_{s}=50$μs, 20 μs) and idealized ($\tau_{s}=0$) switching delays are considered.

Starting with Figure 6, the empirical user outage probability curves for the proposed GeoClust algorithm lie to the right of those corresponding to the benchmark beam placement algorithms. This observation indicates superior outage performance for our GeoClust clustering algorithm. For example, our proposed algorithm can maintain a zero outage probability to all ground users up to a data rate of 1.621 Mbps for K=32 (Figure 6d). This is achieved under the considered heavily congested scenario and $\tau_{s}=50$μs. On the other hand, the LCUD-BPD and the FBD-BP algorithms can offer zero outage up to 0.725 Mbps for K=32. This value is even considerably lower for the PU-BP due to the large switching overhead.

The GeoClust algorithm also achieves significantly improved median user data rates, with the potential to more than double those achieved by benchmark methods. For instance, it achieves a median data rate of 2.821 Mbps for K=32 in Figure 6d. This should be contrasted with 1.279 Mbps for the LCUD-BPD algorithm, 1.0529 Mbps for FBD-BP, and 0.9503 Mbps for PU-BP for the same number of RF chains onboard. Similar trends hold for K=8 and K=16. At K=4, PU-BP performs very poorly due to the high relative impact of beam switching overheads, while the performance gap among GeoClust, LCUD-BPD, and FBD-BP narrows as the system becomes RF resource limited, reducing the impact of cluster formation strategies at the beam-hopping stage.

The key factor that limits the performance of the PU-BP scheme is the beam switching overhead. When the beams are directed to individual users for service (i.e., each ground user is a cluster itself), the system contains too many clusters, and the switching overhead of 50 μs consumes a significant portion of the allocated user time at each beam hop. This leads to inefficient use of available transmission time. More specifically, for K=4 and K=8, it causes a complete outage for all users. With higher numbers of RF chains (e.g., K=16 and K=32), the required number of beam switches decreases and the PU-BP scheme starts to demonstrate better outage performance, but still worse than the other three schemes. These results show the inefficiency of the PU-BP in real beam-hopping scenarios due to the large number of required beam switches.

Figure 7 shows the outage performance of GeoClust and the benchmark algorithms under a reduced beam switching overhead of $\tau_{s}=20$μs. As in Figure 6, GeoClust consistently outperforms all benchmarks. The reduction in switching overhead has a limited effect on GeoClust, LCUD-BPD, and FBD-BP, as these algorithms generate a moderate number of clusters, which do not incur excessive switching delays during the beam-hopping stage.

In contrast, PU-BP, which assigns a dedicated beam per user, experiences a substantial performance gain due to the reduced overhead, particularly at low values of *K*, where switching time was previously a dominant bottleneck. For example, at low data rates, the percentage of users in outage under PU-BP drops from 100% to 12.39% for K=4 and 0% for K=8, indicating the impact of reduced switching overhead. As the number of RF chains increases, the PU-BP algorithm achieves better performance, eventually surpassing LCUD-BPD and FBD-BP, though it continues to underperform relative to GeoClust. It is also important to note that the effectiveness of PU-BP diminishes in less congested scenarios since it inefficiently allocates beams to inactive users. In contrast, LCUD-BPD and FBD-BP apply TDMA within each cluster, ensuring that only active users share the beam resource.

Figure 8 shows the outage performance for the idealized scenario of $\tau_{s}=0$, where the same qualitative trends remain for the proposed algorithm, reflecting those observed in Figure 6 and Figure 7. In general, the performance trends in Figure 6, Figure 7 and Figure 8 demonstrate the ability of our proposed GeoClust algorithm to offer lower outage probabilities by balancing the number and size of the formed clusters. This advantage enables GeoClust to adapt to arbitrary user distributions, enabling efficient integration with the higher-layer beam scheduling and hopping protocols. On the other hand, LCUD-BPD and FBD-BP lack this flexibility and tend to perform well only when the user distribution results in sparsely spaced clusters.

#### 4.4.2. Sum Rate Performance

**Figure 9:**
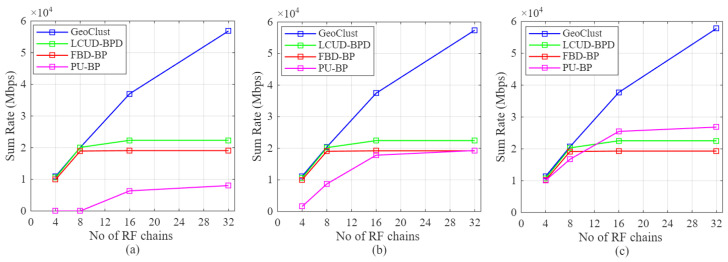
Sum rate performance of the proposed GeoClust, the LCUD-BPD, the FBD-BP algorithms, and the PU-BP scheme for different numbers of RF chains for (**a**) $\tau_{s}=50$μs, (**b**) $\tau_{s}=20$μs, and (**c**) $\tau_{s}=0$.

Next, we evaluate the aggregate downlink throughput achieved by GeoClust against that of LCUD-BPD, FBD-BP, and PU-BP. The results are shown in Figure 9 for three beam switching overheads: $\tau_{s}=50$μs (Figure 9a), $\tau_{s}=20$μs (Figure 9b), and the idealized $\tau_{s}=0$μs (Figure 9c).

As observed in Figure 9, GeoClust offers the highest sum rate across the entire range of RF chain resources ($K\in \left\{4,8,16,32\right\}$), and the performance gap widens as *K* increases. This holds for all the beam switching overheads we consider. The reason for this behavior is that GeoClust can balance cluster size against cluster count, enabling the beam scheduler to achieve highly efficient RF chain utilization when compared to the benchmark schemes. Hence, the beam dwell times, and therefore the aggregate throughput, scale almost linearly with *K* for GeoClust.

This effect is reflected in the scheduler utilization statistics. For the Australian dataset with K=16, GeoClust activates approximately 12.13 beams per hop on average, whereas LCUD-BPD and FBD-BP activate only about 4.93 and 4.78 beams on average, respectively. This indicates that GeoClust achieves substantially higher RF chain utilization under the same distance-based spatial reuse rule, while the benchmark layouts leave a significant fraction of RF resources idle. As a result, GeoClust continues to benefit more effectively from additional RF chains, whereas the benchmark schemes plateau earlier due to resource under-utilization.

This utilization gap also explains the plateauing behavior observed for the LCUD-BPD and FBD-BP schemes. Their cluster layouts require larger spatial separation between simultaneously scheduled beams, which limits the number of beams that can be activated per hop and leads to RF chain under-utilization.

PU-BP exhibits the same flattening, but for a different reason. Although its clusters are points, the minimum inter-beam distance (one beam diameter) sharply limits how many users can be served concurrently once beam footprints start to overlap. Beam footprint overlap occurs under PU-BP in our scenario since the underlying user population is spatially clustered and unevenly distributed, as illustrated in Figure 3.

The sensitivity of PU-BP to switching overhead is also evident in Figure 9. With an overhead of $\tau_{s}=50$μs and K≤8, frequent beam hops consume a large fraction of the beam-hopping window, eroding throughput. Reducing $\tau_{s}$ alleviates this penalty, but PU-BP’s performance still lies below GeoClust because spatial isolation, rather than switching time, becomes the dominant constraint.

An important takeaway from this discussion is that GeoClust sustains throughput growth with additional RF chains and remains robust to switching overheads, whereas all benchmark schemes suffer either from scheduling-induced RF resource idling (LCUD-BPD, FBD-BP) or excessive beam-hopping overhead (PU-BP).

#### 4.4.3. Throughput Histograms

**Figure 10:**
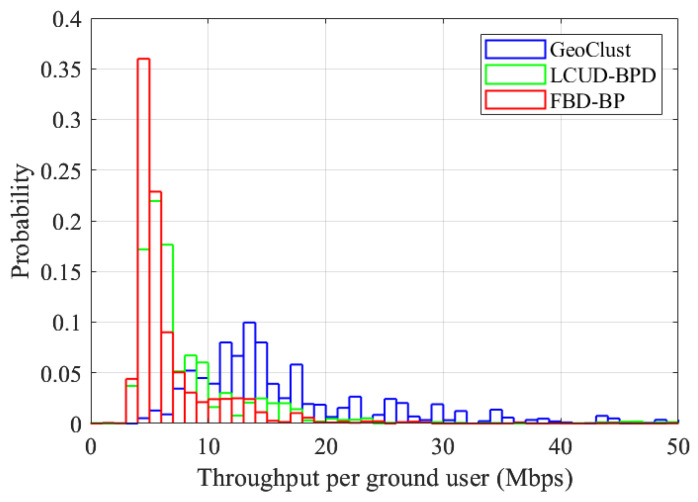
Throughput histograms for the GeoClust, the LCUD-BPD, and the FBD-BP algorithms for K=32 and an activity factor of 20%.

For further investigation, we evaluate the offered per-user data rates in Figure 10. Per-user throughput provides a finer measure of user experience than system-level aggregate data rates and outage curves. For this figure, the beam switching overhead is set to $\tau_{s}=50$ μs, and a 20% user activity factor is assumed (as recommended by 3GPP [59]). We do not include PU-BP in this analysis since, when only a fraction of users are active, the one-beam-per-user approach squanders frame time on inactive locations. The comparison in this section, therefore, focuses on schemes that rely on clustered beam-hopping.

Figure 10 provides the histograms of the offered data rates, computed using (8), for GeoClust, LCUD-BPD, and the FBD-BP under a representative high-capacity configuration with K=32 RF chains. As shown in the figure, the modal throughput bin shifts from 4 to 8 Mbps for the benchmark algorithms to 12–16 Mbps for GeoClust, effectively doubling the data rate experienced by a typical user. In particular, the GeoClust algorithm achieves 14.6 Mbps for the median data rate, whereas the LCUD-BPD and FBD-BP algorithms achieve only 6.39 Mbps and 5.26 Mbps, respectively. Every percentile value for GeoClust in Table 2 is at least 1.8 times higher than LCUD-BPD and FBD-BP statistics.

GeoClust provides markedly stronger performance at both ends of the throughput distribution. At the lower tail (worst case users), the 5th percentile user achieves around 7.4 Mbps, comparable to the median LTE downlink rate. On the other hand, the benchmark schemes achieve 4 Mbps. At the upper tail, GeoClust reaches the 95th percentile throughput exceeding 110 Mbps. This demonstrates our algorithm’s ability to exploit favorable link conditions in sparsely populated beams, an advantage that the benchmark algorithms do not capitalize on.

These results show that GeoClust not only improves aggregate sum rate and outage performance but also lifts the entire per-user throughput distribution. It more than doubles the median service rates and provides 5th percentile user rates almost twice as high as the benchmark schemes, improving both typical and worst-case user experience.

### 4.5. GeoClust Performance Analysis on Other Spatial Datasets

For further investigation, we evaluate the performance of the algorithm using two additional spatial datasets, distinct from the Australian regional population dataset in features and size.

#### 4.5.1. Details of Additional Datasets

**Figure 11:**
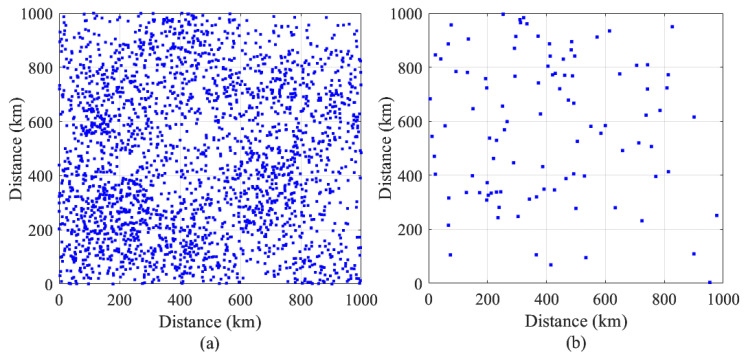
MATLAB-generated user distribution datasets for GeoClust performance analysis (**a**) Dense dataset and (**b**) Sparse dataset.

The additional spatial datasets that we use for further performance analysis are synthetically generated using MATLAB (R2025b), as detailed below:

- **Dataset 1:** A non-uniform spatial dataset consisting of 2500 data points spanning a geographical area of 1000 km × 1000 km. This dataset is generated by first placing population centers uniformly within the geographical area, followed by distributing the data points around each center according to a Gaussian spatial distribution with high variance, resulting in loosely formed clusters with significant spread. The user distribution in this dataset exhibits a slight clustering tendency, reflecting the characteristics of inner regional areas with relatively high populations. This dataset will be referred to as the “dense” dataset in our numerical results below. It is shown in Figure 11a.
- **Dataset 2:** A sparsely distributed dataset comprising 100 data points spanning a geographical area of 1000 km × 1000 km. The user distribution in this dataset reflects the widely dispersed, extremely sparse population characteristics typical of highly remote geographical environments (e.g., polar and arctic regions). This dataset will be referred to as the “sparse” dataset in our numerical results below. It is shown in Figure 11b. We note that the comparison with the PU-BP scheme is not included in the simulations conducted on the “Dense” and “Sparse” datasets below, as its performance is inherently dependent on the dataset size. Given that these datasets are synthetic and their size can be arbitrarily adjusted, including such a comparison yields biased and non-informative results.

#### 4.5.2. User Outage Performance for Dense and Sparse Datasets

**Figure 12:**
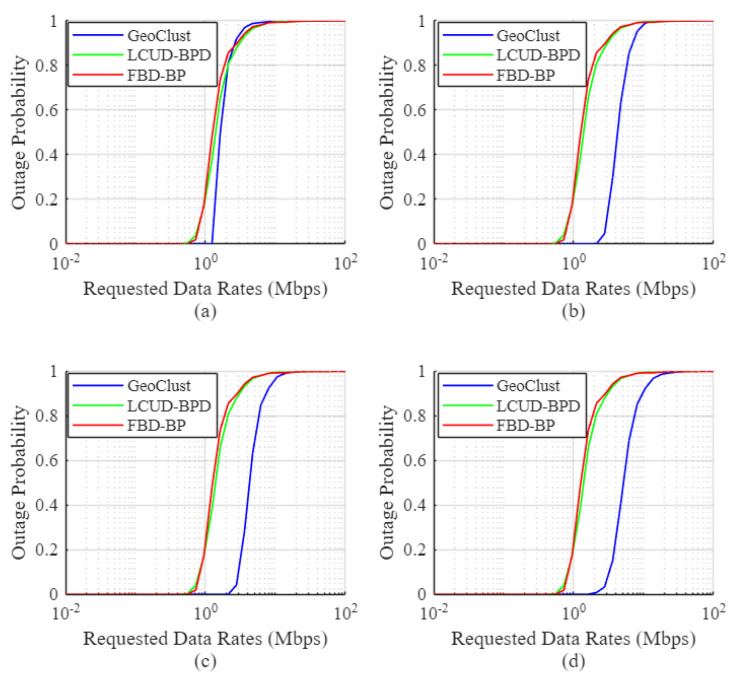
User outage for GeoClust, LCUD-BPD, and FBD-BP for the dense dataset with beam switching overhead ($\tau_{s}=50$ μs) and (**a**) K=4, (**b**) K=8, (**c**) K=16, and (**d**) K=32.

**Figure 13:**
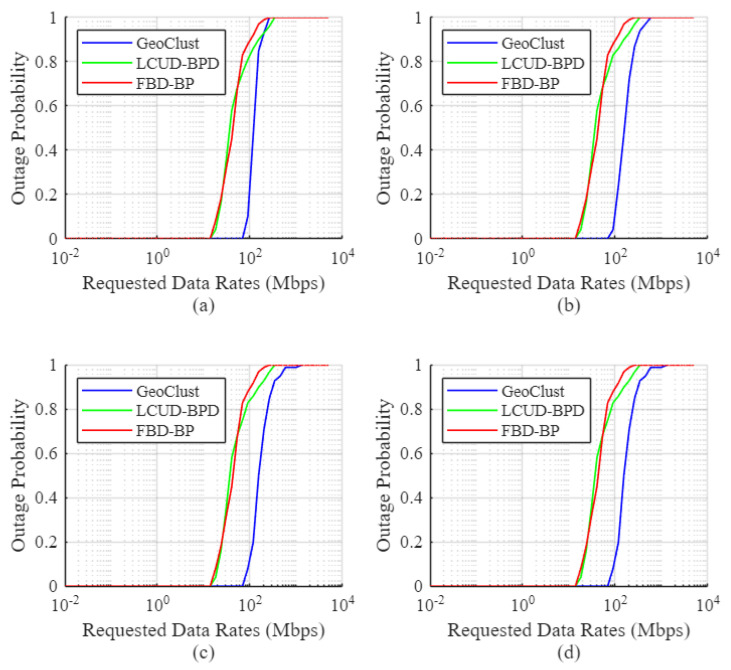
User outage for GeoClust, LCUD-BPD, and FBD-BP for the sparse dataset with beam switching overhead ($\tau_{s}=50$ μs) and (**a**) K=4, (**b**) K=8, (**c**) K=16, and (**d**) K=32. Outage performances of LCUD-BPD and FBD-BP schemes coincide for K=4.

Figure 12 and Figure 13 show the empirical user outage probability of the GeoClust algorithm, in comparison to LCUD-BPD and FBD-BP for the dense and sparse datasets, respectively. The evaluation is conducted under a heavily congested scenario in which all ground users are assumed to be active simultaneously and a beam switching overhead of $\tau_{s}=50$ μs. The outage probability is plotted as a function of the target data rate for different numbers of RF chains *K*. In these figures, beyond K=8, the outage performance improvement becomes marginal because the adopted distance-based beam-hopping scheduler becomes spatial-reuse limited, and consequently, additional RF chains cannot be fully utilized and remain partially idle.

Starting with the dense user distribution, Figure 12 confirms the superior performance of the GeoClust algorithm demonstrated earlier in Figure 6. This performance advantage is evident from the fact that user outage probability curves for the proposed GeoClust algorithm lie to the right of those corresponding to the benchmark beam placement algorithms. More specifically, the GeoClust algorithm can maintain a zero outage probability to all ground users up to a data rate of 2.86 Mbps for K=32 (see Figure 12d). On the other hand, the LCUD-BPD and the FBD-BP algorithms can offer zero outage up to 1.84 Mbps for the same number of RF chains. Moreover, our algorithm provides a median data rate of 5.187 Mbps for K=32 compared to 1.408 Mbps and 1.263 Mbps achieved by LCUD-BPD and FBD-BP, respectively.

Similarly, for the user distribution in the sparse dataset, Figure 13 further reinforces the superiority of the GeoClust algorithm. In this case, the outage probability curves again lie consistently to the right of those for LCUD-BPD and FBD-BP, indicating that GeoClust maintains its advantage even under lighter user congestion. Notably, for K=16, GeoClust sustains a significantly higher median data rate of 152.085 Mbps, achieving more than three times the median data rate of LCUD-BPD (42.541 Mbps) and FBD-BP (37.348 Mbps), confirming that the proposed GeoClust algorithm remains highly efficient even when the user density decreases. We note that the potential advantage of the LCUD-BPD algorithm over FBD-BP scheme is not clearly reflected in these simulations, as the algorithm does not fully exploit the larger user separations inherent in sparse distributions. This limitation arises from the tendency of the cluster radius to increase with the number of users, as demonstrated in [28]. As a result, setting the maximum cluster radius equal to the beam radius leads to a performance that remains close to that of the FBD-BP scheme.

## 5. Conclusions

In this paper, we have introduced a novel geometric user clustering algorithm, *GeoClust*, for multibeam GEO satellite networks. The algorithm addresses a fundamental trade-off between minimizing intra-cluster distances (critical for boosting beam gain) and limiting the number of clusters to control beam-hopping overhead. We have formulated GeoClust as an alternating optimization algorithm with guaranteed convergence to a cluster configuration that satisfies a strict radius feasibility constraint. A central technical contribution is the projection-based constrained center update, implemented via Boyle–Dykstra iterations within the alternating optimization framework. GeoClust runs in polynomial time and admits a tunable structure, where the optimum cluster radius can be determined via a one-dimensional line search over a bounded interval.

For performance evaluation, we have conducted an extensive numerical analysis on a realistic Australian population dataset. The dataset used captures the geographical sparsity and clustering characteristics of regional user distributions, relevant for GEO satellite systems. Our results demonstrate consistent superiority of GeoClust over the benchmark clustering schemes considered in this paper, across a range of performance metrics including outage and throughput.

We have shown that GeoClust achieves more than a twofold increase in both zero outage and median user data rates under a heavily congested load (with 100% user activity) and realistic beam switching delays. We have also shown that the aggregate throughput of GeoClust scales nearly linearly with the number of RF chains. On the other hand, the benchmark algorithms exhibit early saturation due to RF chain under-utilization and spatial multiplexing constraints.

Our analysis of the per-user throughput distribution, conducted under a moderate 20% user activity level, reveals trends consistent with those observed under the heavily congested scenario. GeoClust improves typical user performance by shifting the modal throughput bin from 4 to 8 Mbps for the benchmark algorithms to 12–16 Mbps, more than doubling the median rates. This analysis further shows that GeoClust not only improves performance for typical users, but also enhances service across the entire user population, delivering over 1.8× and 5× gains in the 5th and 95th percentile rates, respectively. These results demonstrate the algorithm’s ability to simultaneously serve both densely populated and sparsely distributed geographical regions.

Our findings carry several important design implications. Firstly, they show that balanced geometry, not simply adding beams, unlocks the true capacity of GEO multibeam systems. Geometry-aware user clustering is vital for fully exploiting RF resources onboard the satellite, under realistic switching delays and heterogeneous traffic distributions. Unlike fixed-beam layouts, GeoClust shows that carefully sized clusters can deliver near-linear throughput scaling and significant improvements in per-user throughput levels. Collectively, these results highlight the importance of incorporating spatial structure and hop-aware trade-offs into the design of next-generation GEO satellite scheduling frameworks.

For future work, several research directions naturally extend the GeoClust framework. First, while GeoClust optimizes clustering under static traffic snapshots, integrating learning-based prediction mechanisms for spatial-temporal traffic evolution can further enhance adaptability in dynamic GEO traffic environments. In particular, data-driven models can be embedded to anticipate demand shifts and proactively adjust cluster geometry and beam placement in real time, enabling predictive rather than reactive resource allocation. Second, the proposed clustering structure provides a principled foundation for advanced physical-layer multiple access strategies. In particular, integrating rate-splitting multiple access within GeoClust-formed clusters can further exploit intra-cluster channel heterogeneity, improving spectral efficiency beyond orthogonal access schemes.

## Figures and Tables

**Table 1:** Simulation parameters.

Parameter	Value
Satellite Altitude (km)	35,786 (GEO)
Frequency band (GHz)	20 (Ka-band)
UPA antenna size	252×252
Transmission bandwidth (MHz)	500
Satellite beam power (watts)	20
Transmit antenna gain (dB)	52
Receive antenna gain (dB)	42
Satellite beam diameter (km)	250
Noise Temperature (K)	290

**Table 2:** Statistical comparison of the offered data rates in Mbps for GeoClust, LCUD-BPD, and FBD-BP algorithms for K=32 and a user activity factor of 20%.

Statistics	GeoClust	LCUD-BPD	FBD-BP
5th Percentile	7.3814	4.0865	4.0252
25th Percentile	11.6435	5.1300	4.5902
50th Percentile	14.6071	6.3952	5.2644
75th Percentile	25.1046	9.5908	7.3489
95th Percentile	113.2214	21.5949	18.2253

## Data Availability

Data are contained within the article.

## Abbreviations

The following abbreviations are used in this manuscript:

ABS	Australian Bureau of Statistics
AWGN	Additive White Gaussian Noise
BH	Beam Hopping
BP	Beam Placement
CCO	Cluster Center Optimization
DVB-S2X	Digital Video Broadcasting-Satellite-Second Generation Extended
FBD	Fixed-Beam Distribution
FBD-BP	Fixed-Beam Distribution Beam Placement
GEO	Geostationary Earth Orbit
GeoClust	Geometric User Clustering Algorithm
GCI	Greedy Cluster Initialization
IBI	Inter-beam Interference
LCUD-BPD	Low-Complexity User Density-Based Beam Placement Design
NP-hard	Non-Deterministic Polynomial-Time Hard
RF	Radio Frequency
RZF	Regularized Zero Forcing
SINR	Signal-to-Interference-plus-Noise Ratio
TDMA	Time Division Multiple Access
UPA	Uniform Planar Array
3GPP	3rd Generation Partnership Project

## Appendix A. Proof of Theorem 1

To prove the theorem, we first re-express the objective function $J\left(\mu \right)=\sum_{i=1}^{n}{∥v_{i}-\mu ∥}^{2}$ according to

(A1) $$J\left(\mu \right)=\sum_{i=1}^{n}{∥v_{i}-\bar{v}∥}^{2}+n{∥\bar{v}-\mu ∥}^{2},$$

where (A1) follows from the standard decomposition of each term ${∥v_{i}-\mu ∥}^{2}$, applying the inner product to ${∥v_{i}-\bar{v}+\bar{v}-\mu ∥}^{2}$ to obtain ${∥v_{i}-\mu ∥}^{2}={∥v_{i}-\bar{v}∥}^{2}+2{\left(\bar{v}-\mu \right)}^{⊤}\left(v_{i}-\bar{v}\right)+{∥\bar{v}-\mu ∥}^{2}$.

Using (A1), the optimization problem in (4) can be equivalently expressed as

(A2) $$\begin{array}{c} \begin{array}{cc} \underset{\mu \in P}{\mathrm{minimize}i}\; & ∥\bar{v}-\mu ∥\\ \mathrm{subject}\;\mathrm{to}\; & \mu \in K \end{array} \end{array}$$

since the first term in (A1) is independent of μ. Therefore, minimizing $J\left(\mu \right)$ over the feasible set K is equivalent to minimizing the distance $∥\bar{v}-\mu ∥$ over K. Hence, the unique minimizer $\mu^{⋆}$ is the projection of $\bar{v}$ onto K, i.e., $\mu^{⋆}=P_{K}\left(\bar{v}\right)$. The uniqueness of $\mu^{⋆}$ follows from the strict convexity of the Euclidean norm.

## Appendix B. Proof of Theorem 2

**Convergence:** Let $J\left(f,{\left\{\mu \right\}}_{i=1}^{f_{\max}}\right)$ be the cost function given in (2). The convergence of the GeoClust algorithm follows from the observation that the Cluster Center Optimization and the nearest-center cluster membership update steps do not increase *J* at each outer iteration of the algorithm. In particular, for fixed cluster memberships, the solution to (4), obtained via Algorithm 1 for each nonempty cluster, yields updated cluster centers that do not increase *J*. Unless the cluster centers are already optimal, this step strictly decreases the first term of *J*. Similarly, for fixed cluster centers, the membership update assigns each ground user to its nearest center. This minimizes the local squared-distance contribution for each user and therefore does not increase the first term of *J*. If the reassignment step produces an empty cluster, this cluster is removed and the remaining clusters are relabeled consecutively, so that $f_{\max}$ is updated to the number of nonempty clusters. Since *g* is non-decreasing, this update cannot increase the second term $\alpha g\left(f_{\max}\right)$.

Therefore, each outer iteration of the GeoClust algorithm produces a nonincreasing sequence of objective values. Moreover, ties in the nearest-center assignment are broken by retaining the current cluster label. Hence any actual change in the clustering rule either strictly decreases the compactness term or reduces $f_{\max}$ through empty-cluster removal. Since the number of clustering rules on *N* users is finite, the clustering rule can change only finitely many times. Once the clustering rule no longer changes, the center-update step returns the same unique centers by Theorem 1, and the algorithm has reached a fixed point. This establishes finite convergence of the outer loop.

**Feasibility Preservation:** Consider the Cluster center optimization problem in (4). This problem is feasible for a given cluster C if the ground user locations for this cluster can be enclosed by a P-ball of radius ρ. By construction, the initial clusters generated by the GCI algorithm satisfy this condition, ensuring feasibility at the first iteration (i.e., t=0) of the algorithm.

During the iterative process, the Cluster Center Optimization step (line 10 of Algorithm 3) updates the cluster centers via projection without altering the cluster memberships. Hence, for each nonempty cluster, all ground user locations assigned to that cluster remain within a distance of ρ from the updated center.

The cluster membership update step (line 13) also preserves feasibility. Each ground user is reassigned to the cluster whose updated center is closest. Since the previous cluster center is one of the candidate centers and the user was already within distance ρ of that center, the distance to the newly assigned center cannot exceed ρ. Therefore, the radius constraint remains satisfied after reassignment. Removing an empty cluster does not affect the feasibility of the remaining clusters.

This concludes the proof because the above arguments show that the Cluster Center Optimization problem, by induction, remains feasible at every iteration of the GeoClust algorithm.
